# Altered Expression of Protamine-like and Their DNA Binding Induced by Cr(VI): A Possible Risk to Spermatogenesis?

**DOI:** 10.3390/biom12050700

**Published:** 2022-05-14

**Authors:** Claudia Moriello, Martina Costabile, Michele Spinelli, Angela Amoresano, Giancarlo Palumbo, Ferdinando Febbraio, Marina Piscopo

**Affiliations:** 1 Department of Biology, University of Naples Federico II, 80126 Naples, Italy; cla_mar97@hotmail.it (C.M.); mart.costabile@studenti.unina.it (M.C.); 2 Department of Chemical Sciences, University of Naples Federico II, Via Cinthia, 21, 80126 Naples, Italy; michele.spinelli@unina.it (M.S.); angela.amoresano@unina.it (A.A.); 3 Commodity Science Laboratory, Department of Economics, Management and Institutions, University of Naples Federico II, 80126 Naples, Italy; gpalumbo@unina.it; 4 Institute of Biochemistry and Cell Biology, National Research Council (CNR), Via Pietro Castellino 111, 80131 Naples, Italy; ferdinando.febbraio@cnr.it

**Keywords:** protamine-like proteins, chromium, *Mytilus galloprovincialis*, stress and protamine-like proteins genes, gonad, spermatozoa, reproduction, sperm chromatin, DNA binding, DNA oxidative damage

## Abstract

Chromium (VI) is the most dangerous oxidation state among the stable forms of chromium. In this work, we evaluated the effect of exposing *Mytilus galloprovincialis* for 24 h to 1, 10, and 100 nM chromium (VI) on the properties of Protamine-like (PLs) and their gene levels in the gonads. Specifically, we analyzed, by AU-PAGE and SDS-PAGE, PLs extracted from unexposed and exposed mussels. In addition, via EMSA, we evaluated the ability of PLs to bind DNA and also verified their potential to protect DNA from oxidative damage. Finally, we assessed possible alterations in gonadal expression of *mt10*, *hsp70*, and genes encoding for PLs-II/PL-IV and PL-III. We found that for all experimental approaches the most relevant alterations occurred after exposure to 1 nM Cr(VI). In particular, a comigration of PL-II with PL-III was observed by SDS-PAGE; and a reduced ability of PLs to bind and protect DNA from oxidative damage was recorded. This dose of chromium (VI) exposure was also the one that produced the greatest alterations in the expression of both *mt10* and *PL-II*/*PL-IV* encoding genes. All of these changes suggest that this dose of chromium (VI) exposure could affect the reproductive health of *Mytilus galloprovincialis.*

## 1. Introduction

Heavy metals are defined as metals with a particular density of greater than 5 g/cm^3^ that have a negative impact on the environment and living organisms [1]. Among heavy metals, chromium (Cr) has become diffused in the environment and can exist in air, water, soil and food [2]. It can be found in different oxidation states. In particular, the trivalent Cr(III) and hexavalent Cr(VI) forms are the forms of Cr that are most thermodynamically stable in nature [3,4]. Cr(VI) has diverse applications in various industries including chemical, refractory, and metallurgical [5]. Of all the ionic forms of Cr, hexavalent chromium [Cr(VI)], is just the most toxic form. It can cross cellular membranes via non-specific anion transporters [6]. Once it has entered the cell, Cr(VI) is reduced to produce reactive intermediates, including Cr(V), Cr(IV), and reactive oxygen species (ROS) [7]. Such species have the potential to induce DNA strand breaks, modification of bases, and lipid peroxidation, destroying cell integrity and causing mutagenic and toxic effects [8]. Chromium (VI) is well known for its detrimental impacts on male reproductive health as a toxic, carcinogenic, and mutagen [9]. Reproductive toxicity of this metal and inhibition of male fertility has been reported in mammals [10]. In addition, in mice severe damage to spermatogenesis following exposure to chromium (VI) [9,11] and the influence of this metal in DNA, RNA, and protein synthesis under sperm-zona binding conditions [12] has been demonstrated. Chromium may also have adverse effects on the reproductive health of marine organisms. As a matter of fact, changes in the concentration of chromium in seawater have been of considerable scientific interest over the past 40 years [13,14,15,16,17]. Although chromium concentrations in seawater are less than 0.5 µg/L, Cr(VI) levels in polluted coastal groundwater caused by Cr-bearing rocks/ores and/or human activities, along with saltwater intrusion, can reach hundreds of µg/L [18]. There are a few studies in the literature regarding the negative effects of chromium exposure on the reproductive health of some marine organisms. From a study conducted on the fish *Carassius auratus*, an upregulation of SOD (Superoxide Dismutase) was observed after chromium exposure, indicating that oxidative stress may be induced by Cr(VI) [5]. In another study performed on the bivalve mollusc, *Galoina coaxsans*, the authors showed alterations in the structure and number of spermatozoa after exposure to Cr(VI) [19]. Accumulation of chromium was observed in the testes of Japanese medaka after exposure to Cr(VI) [20] and in this organism, exposure to Cr(VI) also caused an increase in catalase (CAT) activity and malonyldialdehyde levels in the testes [21]. Finally, pretreatment of gametes with chromium decreases the fertilization capacity of sperm in the sea urchin [22]. In monitoring programs, *Mytilus galloprovincialis* has been widely used as a biological indicator of pollution. This mussel is a sessile, filter-feeding organism capable of accumulating many of the pollutants (pesticides, hydrocarbons, metals, and so on) contained in seawater within its tissues. Mussels also have a wide geographical distribution, which allows these organisms to be studied in large coastal regions [23]. During spermatogenesis in *Mytilus galloprovincialis*, partial replacement of somatic histones by protamine-like (PL) occurs. In the mature spermatozoa, three PL (PL-II, PL-III and PL-IV) are present in this organism [24]. These proteins are essential for the *Mytilus galloprovincialis* sperm chromatin structuring as they account for 76% of the nuclear basic protein components [24]. Since subtoxic doses of metals such as copper, cadmium, and mercury have already been shown to induce alterations in *Mytilus galloprovincialis* PL [25,26,27,28,29,30,31,32], in the present work, we aimed to evaluate the possible effects of chromium (VI) on the properties of *Mytilus galloprovincialis* PL by exposing mussels to 1, 10, and 100 nM Cr(VI). We chose these exposure doses considering that the concentration of total dissolved chromium in seawater and open-ocean surface waters are about 0.1–0.55 and 0.1 × 10^ −3^–0.55 × 10^−3^ μg L^ − 1^, respectively, and usually decreases with depth [33]. Therefore, a potential risk to the fertility of this organism and probably also to many other marine invertebrates could be posed by any detectable alteration in the properties of this type of proteins, which are essential for the correct structure of the sperm chromatin of *Mytilus galloprovincialis*. To assess this, after exposure of mussels to these three doses of chromium (VI), we extracted PLs from spermatozoa and analyzed them by polyacrylamide-acetic acid gel electrophoresis (AU-PAGE) and SDS-PAGE to reveal changes in the electrophoretic pattern. In addition, by EMSA, we assessed the ability of PLs to bind DNA and determined their potential to protect DNA from oxidative damage. Finally, we evaluated, by RTqPCR, the possible alterations in the expression of gonadal *hsp70*, *mt10*, *PL-II*/*PL-IV,* and *PL-III* genes following exposure of mussels to chromium (VI), given that other heavy metals have been shown to cause alterations in some stress genes and in those encoding Protamine-like PL-II and PL-IV [25,34]

## 2. Materials and Methods

### 2.1. Ethics Statement

This research was performed on the marine invertebrate *M. galloprovincialis* (Lamarck, 1819), which is not protected by any environmental agency in Italy. This study was conducted in strict accordance with European (Directive 2010/63) and Italian (Legislative Decree n. 116/1992) legislation on the care and use of animals for scientific purposes.

### 2.2. Mussels Sampling and Exposure to Cr(VI)

*M. galloprovincialis* adult mussels were provided by Eurofish Napoli S.R.L. Baia, in Naples, and used in this research. The mussel had medium size of the shell length 4.93 ± 0.17 cm. Fifteen mussels of undetermined sex were exposed to Cr(VI) concentrations of 1, 10, and 100 nM; a standard aqueous solution of K_2_Cr_2_O_7_ 50.0 mM was used to realize these exposure doses. Mussels were exposed in laboratory plastic tanks (36 cm × 22 cm × 22 cm) holding 6 L of 33‰ artificial sea water (ASW) with the following composition for 1 L: NaCl 29.2 g, KCl 0.60 g, MgCl_2_ 1.2 g, NaHCO_3_ 0.20 g, and CaCl_2_ 1.08 g, as previously stated for the experiment conducted with other heavy metals [26]. Mussels were given a single dosage of Cr(VI) in each tank. The exposure was carried out at 18 ± 1 °C for 24 h, with water and metal salts being changed every 12 h and dissolved oxygen and temperature being recorded at specified intervals. The tests were carried out in March of last year. Tanks containing only ASW were utilized as a control for unexposed mussels.

### 2.3. Gonad Sampling and PL Proteins Extraction

After the exposure for 24 h to the three different concentrations of Cr(VI), mussels were opened by forcing the valves with the use of a knife, being careful not to cut the soft tissues. Mussel’s sex was identified by gonad and gametes examination under a light microscope. The sex of the mollusk was identified by observation of the gametes under a light microscope. Gametes were obtained after stimulation of the male gonads with a Pasteur pipette and the use of seawater, as previously described in Piscopo et al., 2018 [32]. In brief, the semen collected from all the male mussels contained in the tanks corresponding to a specific condition were pooled and centrifuged at 1000× *g* for 2 min at 4 °C to remove debris. In order to collect the spermatozoa, the supernatant obtained was centrifuged at 9000× *g* for 10 min at 4 °C. The sperm-containing pellets of approximately 200 mg were recovered and stored at −80 °C for further investigation. Gonads of male organisms were stocked and stored at −80 °C. Spermatozoa were used for acid extraction of PL proteins. In particular, PL proteins were extracted from spermatozoa using 5% perchloric acid (PCA), as described earlier in Notariale et al., 2018 [35]. For this study, n = 10 spermatozoa pellets from each tank corresponding to a certain nM Cr(VI) condition were used. PCA was added after that the spermatozoa pellets were homogenized in a potter with 15 mL of distilled water as described in Vassalli et al., 2015 [24], and the suspension was kept at 4 °C under stirring for 16 h. At the end, the samples containing PCA-soluble PL-proteins were dialyzed against distilled water to ensure that all PCA was eliminated. The proteins obtained were then lyophilized and kept at a temperature of −80 °C.

### 2.4. Plasmid DNA Preparation

In order to obtain high amounts of supercoiled pDNA, the pGEM3 plasmid (2867 bp) was extracted from transformed *Escherichia coli* HB 101 cells using the standard protocol of the QIAGEN Plasmid Midi Purification kit (QIAGEN Plasmid Midi Purification handbook, third edition 2020, Hilden, Germany), but with the precautions explained in Carbone et al., 2012 [36]. Gel electrophoresis on 1% agarose gels in 89 mM Tris-HCl pH 8.0, 2 mM EDTA, and 89 mM boric acid (TBE) was used to analyze the plasmid DNA. For electrophoretic mobility shift assays (EMSA) of DNA and study of DNA breakage/protection by PLs, the circular form of the plasmid pGEM3 was employed.

### 2.5. Extraction of Sperm DNA

Sperm DNA was extracted from spermatozoa of *Mytilus galloprovincialis* specimens unexposed to metals, as described in Piscopo et al., 2018 [32]. In brief, 200 mg of spermatozoa pellet were resuspended in 500 µL of 2× lysis buffer (20 mM Tris-HCl pH 8.0, 20 mM EDTA pH 8.0, 200 mM NaCl, 4% SDS, 20 µg/mL Proteinase K). The sample was incubated for 30 min at 37 °C before being centrifuged for 15 min at 4 °C at 15,000 rcf. The pellet was rinsed in 1× lysis buffer, and then the sample was centrifuged under the same conditions. The pellet obtained was suspended in 1× lysis buffer with 39 mM DTT and stored at 37 °C overnight. Phenol/chloroform extraction and ethanol precipitation were used to get DNA. A TE buffer (1 mM EDTA, 0.01 M Tris-HCl pH 8.0) was used to suspend purified DNA.

### 2.6. Electrophoretic Analysis

To analyze protein samples, two types of electrophoretic analyses were used: AU-PAGE, and SDS-PAGE as earlier described by Piscopo et al., 2018 [37] and by Piscopo et al., 2020 [38], respectively, with few improvements. Below is the gel recipe of 15% polyacrylamide gel electrophoresis in urea acetic acid (AU-PAGE): 15% Acrylamide/Bisacrylamide (starting from Acrylamide/Bis-acrylamide 30:0.2); 2.5 M urea; 5% Acetic Acid; 0.75% Temed; 0.15% APS. For the SDS-PAGE, the stacking gel was constituted by 5% (*w*/*v*) acrylamide (acrylamide/bis-acrylamide 30:0.15), and the separating gel was 18% (*w*/*v*) acrylamide (acrylamide/bis-acrylamide 30:0.15). The gels were stained with Coomassie Brilliant Blue at the end of both AU-PAGE and SDS-PAGE. Quantity One v.4.4.0 (BioRad, Hercules, CA, USA) software was used to acquire gels using a Gel-Doc system (BioRad, Hercules, CA, USA). Densitometric analysis on gel bands was conducted using the software ImageJ ver. 1.50 d (https://imagej.nih.gov/ij/ (accessed on 7 March 2022)) supported by the National Institute of Health (Wayne Rasband, National Institute of Mental Health).

### 2.7. Analysis of the Effect of M. galloprovincialis PL Proteins on DNA Electrophoretic Mobility

The effect of PL proteins of *M. galloprovincialis* after exposure to Cr(VI) on DNA Electrophoretic Mobility was analyzed by Electrophoretic Mobility Shift Assay (EMSA). The procedure published [24] was followed with some modifications. In particular, in all samples, a fixed amount of plasmid DNA (pGEM3) (150 ng) and increasing amount of PLs were used in order to obtain protein/DNA wt/wt ratios between 0.1 and 1.8, as indicated in the section results. We looked at the protein/DNA ratio that was required to achieve DNA saturation, i.e., the condition in which DNA is present as unique band close to the well. The following ingredients were added to the samples in the following order: ultrapure water (milliQ), DNA, and proteins, for a final volume of 30 µL. The samples were then left at room temperature for 5 min to allow the proteins and DNA to interact. After the interaction, all samples were added with TBE 10× (in order to obtain TBE 1× final concentration) just before running the gels and analyzed on 1% agarose gel in TBE 1× final concentration. Electrophoresis was carried out at 100 V for 30 minutes. The EMSA was also conducted using genomic DNA extracted from spermatozoa of *Mytilus galloprovincialis* unexposed (sperm DNA). The modality of preparation of the same was similar to that for plasmid DNA with the difference that a fixed amount of genomic DNA (450 ng) and increasing amounts of PLs were used for a range of wt/wt protein/DNA ratios from 0.2 to 0.5. The electrophoretic run was performed on a 0.6% agarose gel at 60 V for approximately 1 h. After electrophoresis, gels were stained with ethidium bromide (2 mg/mL) to observe DNA migration. Quantity One v.4.4.0 (BioRad, Hercules, CA, USA) software was used to collect gels using a Gel-Doc system (BioRad, Hercules, CA, USA). The program ImageJ ver. 1.50 d (Wayne Rasband, National Institute of Health, Bethesda, ML, USA, https://imagej.nih.gov/ij/ (accessed on 7 March 2022), 1997–2018) was used to do a densitometric analysis of the bands on the gel.

### 2.8. DNA Protection Analysis

PLs ability to protect DNA from oxidative damage in the presence of 30 µM H_2_O_2_ and 5 µM CuCl_2_ was evaluated by using plasmid DNA (pGEM3) and PLs extracted from the spermatozoa of mussels exposed at the three different concentration of Cr(VI). The samples were prepared using EMSA protocol described in the paragraph “Analysis of the Effect of *M. galloprovincialis* PL Proteins on DNA Electrophoretic Mobility”, with slight modifications. In particular, 150 ng of plasmid DNA (pGEM3) and proteins/DNA wt/wt ratios in a range from 0.4 to 0.8 were used. H_2_O_2_ and CuCl_2_ were added after 5 min of room temperature interaction between DNA and PLs, and the samples were incubated for 30 min in the dark in a Thermoblock set at 37 °C. To minimize EDTA coordination of subsequent metals, sample buffer 10× (1× final concentration in the samples) was added right before electrophoresis analysis at the conclusion of incubation. The electrophoretic analysis of the samples was conducted on 1% agarose gel at 100 V for 30 min in TBE 1×. The assay was also conducted using genomic DNA extracted from spermatozoa of *Mytilus galloprovincialis* unexposed (Sperm DNA) in the presence of 70 µM H_2_O_2_ and 50 µM CuCl_2_, using a fixed amount of genomic DNA equal to 450 ng and increasing amount of protein considering the different wt/wt protein/DNA ratios. A range of ratios between 0.4 and 0.8 was used. Samples were loaded on 0.6% agarose gels. The run was performed at 60 V for 1 h in 1× TBE buffer. After electrophoresis, agarose gels were stained with ethidium bromide (2 g/mL) to observe DNA migration, and the gels were acquired using the GelDoc Biorad (Hercules, CA, USA).

### 2.9. RNA Extraction, cDNA Synthesis and RT-qPCR

Total RNA was isolated from the gonads of unexposed mussels (control) and exposed to 1, 10, and 100 nM Cr(VI) using Trizol reagent (Invitrogen, Carlsbad, CA, USA), as indicated by the manufacturer. A UV-Vis spectrophotometer was used to determine the quantity of RNA extracted (NanoDropH ND-1000, Waltham, MA, USA) (NanoDropH ND-1000, Waltham, MA, USA). An electrophoretic analysis was done on 1% agarose gels under denaturing conditions to determine the quality of RNA. The DNA-free kit from Ambion (Austin, TX, USA) was used to eliminate genomic DNA from the samples. M-MLV reverse transcriptase was used to obtain cDNA from 1 µg of RNA from each sample (ImpProm II kit, Promega, Madison, WI, USA). The RT-qPCR was carried out as described in Lettieri et al., 2021 [30]. To evaluate gene expression, the 7500 Real Time PCR System was used with 100 ng of cDNA and 10 µM of each forward and reverse primer in a final volume of 50 µL using the SYBRGreen PCR Master Mix Kit (Applied Biosystems, Foster City, CA, USA) (Applied Biosystems, Foster City, CA, USA). As indicated in Lettieri et al., 2019 [25], each PCR reaction was run for 40 cycles, maintaining the following conditions: Denaturation at 95 °C for 15 min; annealing and elongation at 60 °C for 1 min. The primers that were utilized are listed in Table 1. The open-source program Primer3 was used to design the reaction primer, which was based on the sequences and accession numbers shown in Table 1. The findings were exported from the ViiA™-7 Software into Microsoft Excel (Redmond, WA, USA, ver. 2009—build 13,231.20262). (Foster City, CA, USA). The ∆∆Ct methodology, as described by Livak e Schmittgen, 2001 [39], was used to estimate the relative quantification of gene expression. The expression of the genes *mt10*, *hsp70*, *PL-III*, and *PL-II*/*IV* in mussels was compared to that of control mussels.

### 2.10. Determination of the Sperm Motility

Sperm motility was assessed using sperm suspensions prepared by diluting the sperm collected from the mantle in ASW (1:100 *v*/*v*). Five subjective motility score classes were estimated: class 1 corresponded to no motility to 20% motility, class 2 from 20 to 40% motility, class 3 from 40 to 60% motility, class 4 from 60 to 80% motility, and class 5 to maximum motility (from 80 to 100% motility) [40,41]. A sample of 10 µL of each sperm suspension was put on a slide, covered with a coverslip, and evaluated with a Zeiss microscope by using a 40× objective..

### 2.11. Fluorescence Spectroscopy of PL Proteins

The fluorescence analysis was carried out in a 0.5 mL volume cuvette (1 cm optical path length) (STARNA) using a Jasco spectrofluorimeter model FP 8200, using 8-anilino-1-naphthalenesulfonic acid (ANS) as a probe. Fluorescence emission of ANS (5 μM) in absence and in presence of 0.01 or 0.02 mg/mL concentration of PLs in H_2_O milliQ was performed in the range from 420 to 600 nm after excitation at 350 nm. Spectra were acquired in presence of increasing concentrations of chromium (VI) in the range from 0 to 100 nM using 0.02 mg/mL concentration of PLs in H_2_O milliQ. Each spectrum was signal averaged at least three times and smoothed with the software Spectra Manager ver. 2.09 (Jasco Corporation, Tokyo, Japan). All measurements were performed at least three times at room temperatures.

## 3. Results

### 3.1. Electrophoretic Analysis of PL Proteins

**Figure 1:**
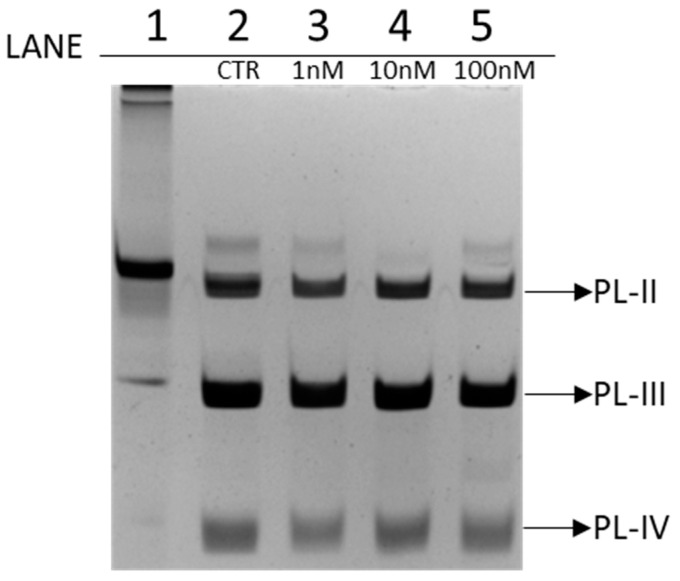
AU-PAGE of PLs extracted from mussels. Lane (1) *Chaetopterus variopedatus* sperm H1 Histone; lane (2) PL extracted from unexposed mussels (CTR, ie control); lanes (3–5) PL extracted from mussels exposed to 1, 10, and 100 nM chromium (VI), respectively. CTR = unexposed mussels.

PL proteins extracted from spermatozoa of *Mytilus galloprovincialis* exposed to 1 nM, 10 nM, and 100 nM chromium (VI) were analyzed by AU-PAGE and SDS-PAGE to evaluate possible differences in the electrophoretic pattern compared to PLs extracted from spermatozoa of unexposed mussels. 4 µg of proteins were loaded into each well. By AU-PAGE, shown in the Figure 1, PL-II, PL-III, and PL-IV can be observed from top to bottom. In particular, in lane 1 was loaded *Chaetopterus variopedatus* sperm H1 histone, in lane 2 were loaded PLs extracted from unexposed mussels (CTR), from lane 3 to 5 were loaded PLs extracted from exposed mussels to 1 nM, 10 nM, and 100 nM chromium (VI), respectively.

AU-PAGE (Figure 1) showed no significant differences in the electrophoretic pattern between PL extracted from exposed and unexposed mussels.

**Figure 2:**
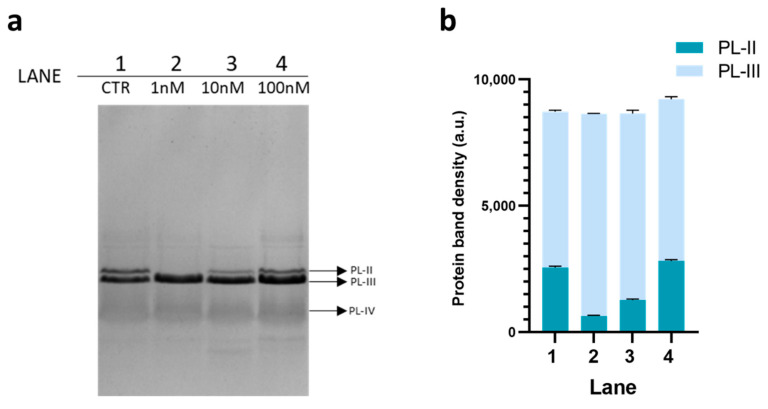
SDS-PAGE (panel (**a**)) and densitometric bands analyses (panel (**b**)) of PLs extracted from mussels. Lane (1) PLs extracted from unexposed mussels (CTR); Lane (2–4) PLs extracted from mussels exposed to 1, 10 and 100 nM chromium (VI), respectively. CTR = unexposed mussels.

On the same samples showed in Figure 1 SDS-PAGE analysis was also conducted (Figure 2).

For this analysis, 6 µg of PLs were loaded on the gel for each condition. As in the previous gel, PLs extracted from unexposed mussels (CTR) were loaded in lane 1 (panel a, Figure 2), whereas in lane 2 to 4 were loaded PLs extracted from mussels exposed to the three doses of chromium (VI). In this case, the three proteins were visible in the (CTR): from top to bottom PL-II, PL-III, and PL-IV. PL-II and PL-III having very close molecular weight values (13.8 and 11.8 kDa, respectively) exhibit very similar electrophoretic mobility. The main differences observed with this type of electrophoretic analysis was a reduction of the amount of PL-II in the canonical mobility position, caused by a comigration with PL-III. In mussels exposed to 1 nM chromium (VI) compared to unexposed mussels, a total comigration of PL-II with PL-III was observed; in mussels exposed to 10 nM chromium (VI), only a fraction of PL-II comigrates with PL-III, whereas in mussels exposed to 100 nM chromium (VI), a condition similar to unexposed mussels seemed to be re-established.

### 3.2. Analysis of PL-DNA Interaction by EMSA

#### 3.2.1. EMSA with Plasmid DNA

**Figure 3:**
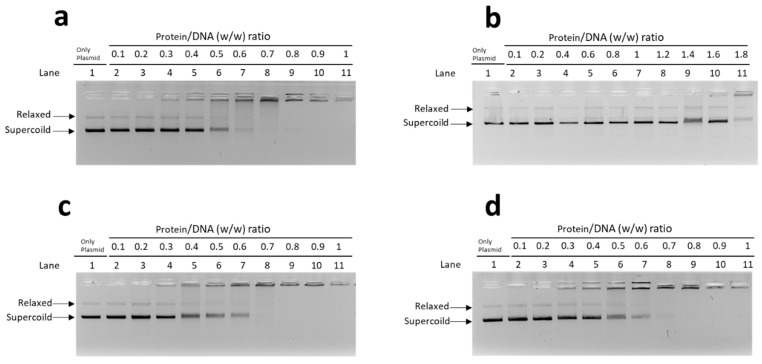
EMSA with: (**a**) PLs from unexposed mussels; (**b**) PLs extracted from mussels exposed to 1 nM chromium (VI); (**c**) PLs extracted from mussels exposed to 10 nM chromium (VI); (**d**) PLs extracted from mussels exposed to 100 nM chromium (VI). Only plasmid denotes plasmid DNA without proteins; relaxed = relaxed DNA plasmid; supercoiled = supercoiled DNA plasmid.

In these EMSA, a fixed DNA concentration (150 ng) and PL/DNA ratios (wt/wt), ranging from 0.1 to 1.8, were used (Figure 3). The protein-to-DNA ratio at which DNA reaches saturation was evaluated. By “saturation”, we mean the condition in which the DNA is complexed with the protein in the form of a single band near to the well, and therefore neither the band corresponding to supercoiled DNA nor that of relaxed DNA is more visible.

In the assay conducted with PLs extracted from unexposed mussels, as shown in panel a, the DNA saturation was reached at a wt/wt Protein/DNA ratio of about 0.8. A similar situation was observed for the PL from mussels exposed to 10 nM and 100 nM chromium (VI), (c and d, respectively). Differently, saturation was not reached even at very high ratios of wt/wt Protein/DNA (1.8) with PL from mussels exposed to 1 nM chromium (VI) (panel b).

#### 3.2.2. EMSA with Sperm DNA

**Figure 4:**
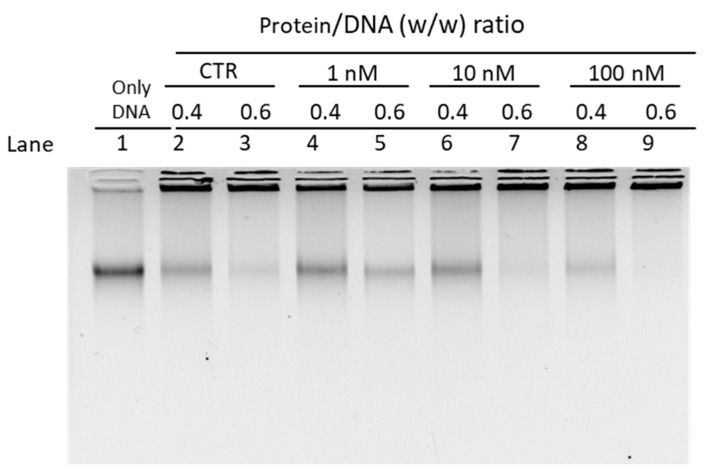
EMSA with sperm DNA conducted with PLs from unexposed mussels (CTR) and exposed to 1 nM, 10 nM and 100 nM chromium (VI). CTR = unexposed mussels.

For the EMSA performed with sperm DNA a fixed amount of sperm DNA (450 ng) and increasing amounts of PL from mussels unexposed and exposed to the three different concentrations of chromium (VI), in order to reach wt/wt protein/DNA ratios ranging from 0.4 to 0.6. (Figure 4).

Under all four conditions, almost complete DNA saturation at the w/w protein/DNA ratio 0.6 was observed both for unexposed mussels (lane 3) and in mussels exposed to 10 and 100 nM chromium (VI) (see lanes 7 and 9). In contrast, for mussels exposed to 1 nM chromium (VI), the fraction of saturated DNA was lower, and a greater fraction of DNA resulted in high mobility compared to previous conditions (see lane 5).

### 3.3. Analysis of the Ability of PLs to Protect DNA from Oxidative Damage

**Figure 5:**
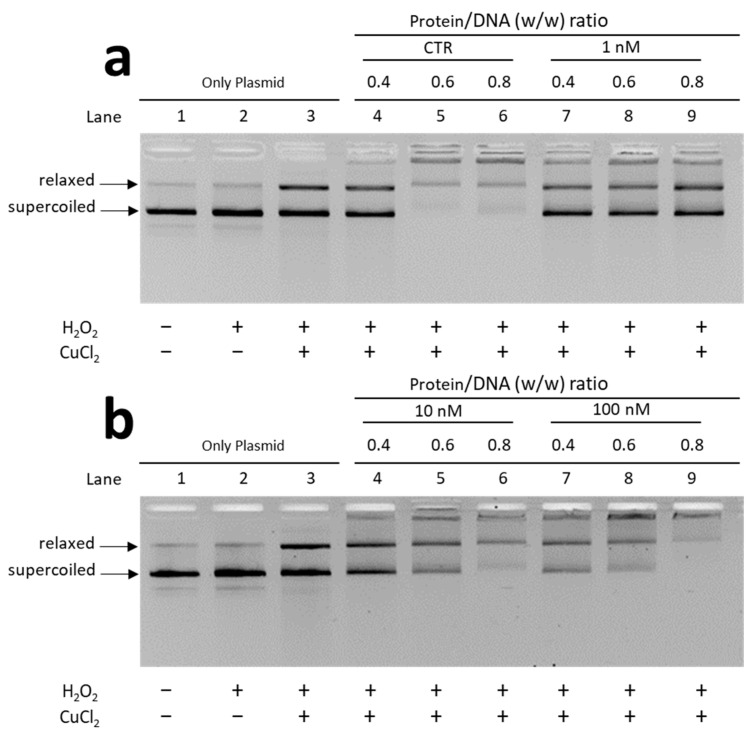
Protection assays with PL extracted from: unexposed (CTR) and exposed mussels to 1 nM chromium (VI) (**a**); exposed to 10 nM and 100 nM chromium (VI) (**b**). CTR = unexposed mussels.

Given the differences in the electrophoretic pattern of PLs from mussels exposed and unexposed to chromium and considering the changes in the affinity of PLs for DNA, we evaluated the ability of these proteins to protect DNA from oxidative damage. DNA damage was assessed by means of a protective assay, under pro-oxidant conditions, i.e., in the presence of appropriate concentrations of H_2_O_2_ and CuCl_2_ as specified in Materials and Methods in order to cause the Fenton reaction. The experiments were conducted using both plasmid DNA and genomic DNA extracted from unexposed mussels (sperm DNA). Below we first show the results obtained using plasmid DNA (Figure 5)

In the wells indicated with number 3 of the gels, is shown the result obtained from a DNA damage condition that we induced by treating the DNA at appropriate concentrations of H_2_O_2_ and CuCl_2_. In this condition the oxidative DNA damage is revealed by the fact that the band corresponding to the relaxed DNA appears more intense than the band of the relaxed DNA in the sample in which only DNA and H_2_O_2_ were inserted (wells number 2). The effects on DNA of adding PLs to the mixture composed by DNA, H_2_O_2_ and CuCl_2_ were evaluated for both exposed and unexposed mussels at *w*/*w* protein/DNA ratios of 0.4, 0.6 and 0.8.

**Figure 6:**
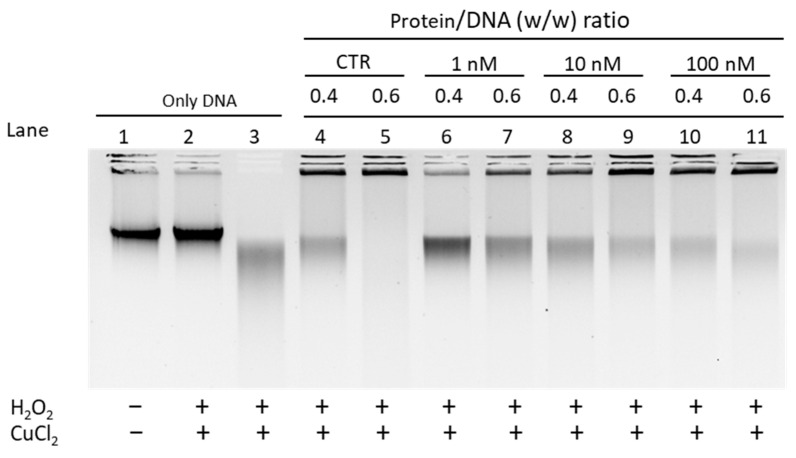
Protection assay conducted with sperm DNA and PL extracted from unexposed and exposed mussels to 1 nM, 10 nM, and 100 nM chromium (VI). CTR = unexposed mussels.

The assay performed with PLs from unexposed mussels reveals that these proteins have DNA protective capacity as can be seen in samples at protein/DNA ratios of 0.6 and 0.8 (panel a, lanes 5 and 6). Under these conditions, the DNA does indeed appear organized with the proteins being mostly near the well, and the band of relaxed DNA is very light and no more supercoiled DNA is observed. A trend towards DNA protection is also observed for PLs from mussels exposed to 10 and 100 nM chromium (VI) (see panel b lanes 5–6–8–9), although less pronounced than for PLs from unexposed mussels. In contrast, no protection against DNA is revealed for PLs from mussels exposed to 1 nM chromium VI (panel a, lanes 7 to 9). Indeed, in this condition, the addition of PLs do not significantly change the electrophoretic pattern of plasmid DNA compared to that shown in well 3. The protective assay performed by using sperm DNA from unexposed mussels gave the results shown in Figure 6.

Additionally in this case, a condition of damage was purposely generated for the spermatic DNA, which, as can be seen in well 3, appears degraded. For sperm DNA, only the addition of PL from mussels unexposed and exposed to 10 and 100 nM chromium (VI), to the mixture containing sperm DNA, H_2_O_2_, and CuCl_2_ allowed almost complete DNA protection, at the 0.6 protein/DNA ratio (lanes 5, 9, and 11). In contrast, PL extracted from mussels exposed to 1 nM chromium (VI) (see lanes 6 and 7) showed a lower capacity to protect DNA from oxidative damage; in fact, at protein/DNA ratio of 0.4 most of the DNA is degraded (lane 6) and DNA protection obtained at 0.6 protein/DNA ratio is lower than previous conditions (well 7).

### 3.4. Gonadal mt10, hsp70 and PL-Proteins Genes Expression

**Figure 7:**
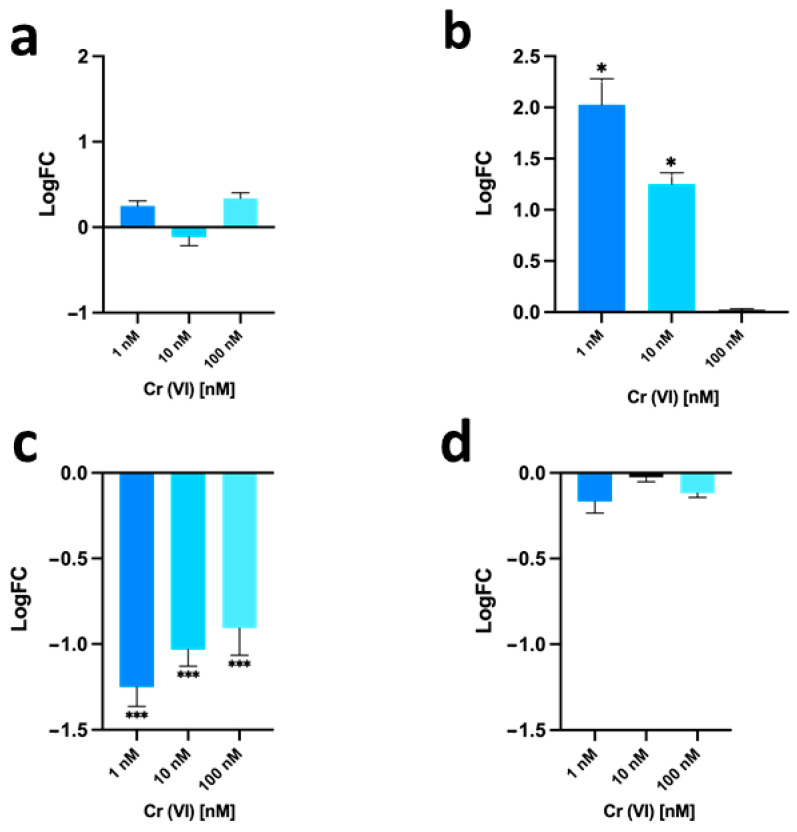
RT-qPCR of *hsp70*, *mt10*, *PL-II*/*PL-IV* and *PL-III* in *M. galloprovincialis* gonads. In the figure, the change in expression of *hsp70* (**a**), *mt10* (**b**), *PL-II*/*PL-IV* (**c**), and *PL-III* (**d**) is reported under the three chromium (VI) exposure conditions compared to the control condition (unexposed mussels). Expression was determined with respect to the housekeeping gene *GAPDH*. Values are presented as mean ± S.D. (n = 6). Asterisks indicate a statistically significant difference from unexposed mussels: * = *p* < 0.05; *** = *p* < 0.001.

Quantitative polymerase chain reaction with reverse transcription (RT-qPCR) was used to evaluate the expression of some genes as a response to possible stress. The gene expression level of the stress genes *hsp70* and *mt10* in the gonads of mussels exposed to the three different concentrations of chromium (VI) (1, 10, and 100 nM) and of the genes encoding for PL-II/PL-IV and PL-III were evaluated (Figure 7).

For the *hsp70* gene (see panel a), no significant changes in expression were observed in the three different exposure conditions compared to unexposed mussels. For the *mt10* gene (see panel b), there was an increase in expression of two-fold and about one-fold following 1 nM and 10 nM chromium (VI) exposure, respectively, compared to unexposed mussels. In mussels exposed to 100 nM chromium (VI), instead no change in expression was observed compared to unexposed mussels. For the *PL-II*/*PL-IV* gene (see panel c), a down-regulation of expression was observed after exposure, which was more relevant in the 1 nM chromium (VI) exposure condition, resulting in about 1.3-fold lower than in unexposed mussels. For the *PL-III* gene (see panel d) for all exposure conditions no significant changes in expression were observed compared to unexposed mussels.

### 3.5. Evaluation of Sperm Motility

Given the alterations found at protein and gene level after the exposure of mussels to chromium (VI), we evaluated the sperm motility. In Table 2, mean values of sperm motility score are reported. Measurements were made on sperm suspensions from *M. galloprovincialis* unexposed and exposed to 1, 10, and 100 nM chromium (VI). For all the exposure doses, a reduced number of motile spermatozoa was observed. In particular, it is interesting to note that just following 1 nM chromium (VI) exposure, the number of motile spermatozoa was very low in comparison with unexposed and exposed mussels to 10 and 100 nM chromium (VI). In addition, always at this exposure dose, the lowest score was recorded. The exposure at 10 and 100 nM chromium (VI) produced the same score (3).

### 3.6. Fluorescence Spectroscopy of PL Proteins in ANS

**Figure 8:**
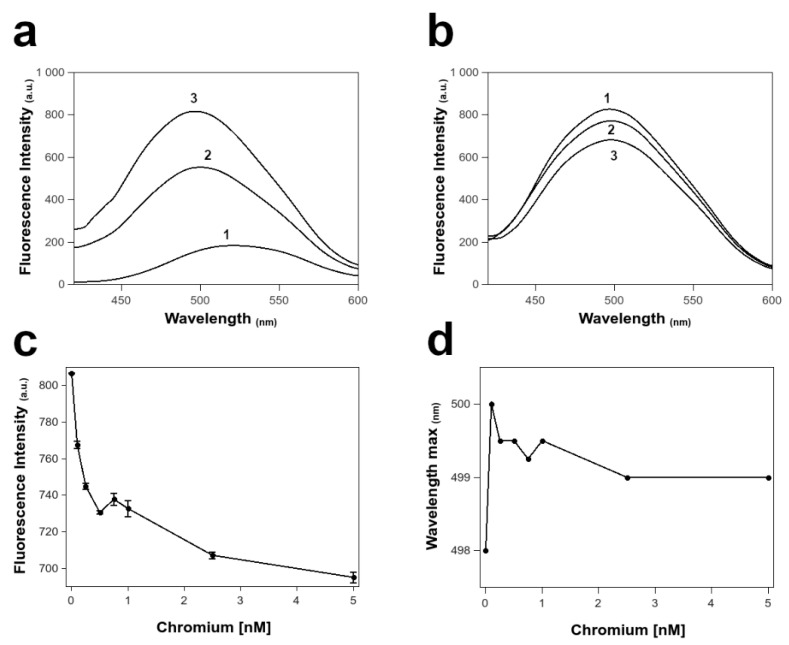
ANS-PLs fluorescence analysis. (**a**) Spectra of ANS fluorescence emission alone (1) and in presence of PLs at (2) 0.01 mg/mL and (3) 0.02 mg/mL. (**b**) Spectra of ANS fluorescence emission in presence of PLs at increasing concentration of chromium (VI) (1 = 1 nM, 2 = 10 nM, 3 = 100 nM). (**c**) Plot of the maximum fluorescence intensity values of ANS in presence of PLs at increasing concentration of chromium (VI) in the range from 0 to 5 nM. (**d**) Plot of the maximum fluorescence emission wavelength of ANS in presence of PLs at increasing concentration of chromium (VI) in the range from 0 to 5 nM. All measurements in (**c**,**d**) were performed at least three times at room temperatures.

In the presence of the three PLs mixture, we observed a fluorescence increase and a blue shift in the maximum of ANS fluorescence emission from 521 to 498 nm (Figure 8a), indicating the binding of the probe to the PLs. Addition of increasing concentration of chromium (VI) to the ANS bound PLs complex reduced the emission intensity along with a little red shift of the emission maximum (Figure 8b). The plot of the maximum fluorescence intensity values of the ANS in presence of PLs at increasing concentration of chromium (VI) in the range from 0 to 5 nM (Figure 8c), indicated a change in the interaction between the ANS and the PLs mixture between the concentration of 0.5 and 1 nM of the metal. This event is confirmed by the same change in the red shift in the wavelength of the emission maximum in the probe fluorescence at these metal concentrations (Figure 8d).

## 4. Discussion

Reproductive success is a key determinant for the survival of the species. In the last decade, the greatest risks affecting reproduction success occur for those species that are externally fertilized, such as several marine invertebrates, since many marine ecosystems have been contaminated with different types of xenobiotics. This is because the process of external fertilization is extremely susceptible to marine xenobiotics in seawater resulting from industrial and agricultural activities [42]. External fertilization, in fact, involves the release of sperm and eggs into seawater, which aids in the locomotion of sperm. Therefore, both gametes and the resulting larvae are at risk of exposure to contaminants, and a number of marine invertebrates, living and breeding in coastal areas polluted by various kinds of chemicals, can be affected at different levels of biological organization [43]. A relevant role among seawater contaminants is played by heavy metals, whose release into the marine environment has largely increased their levels in recent decades, accumulating in bottom sediments and consequently in marine organisms [44,45]. Among the various heavy metals, chromium has attracted special attention as it is a toxic metal present in marine water and groundwater due to natural and anthropogenic sources [46]. Chromium is found in the aquatic environment in predominantly two forms, trivalent chromium (Chromium III or Cr^3+^), which occurs naturally, and hexavalent chromium (Chromium VI or Cr^6+^), which also occurs naturally due to erosion of chromium deposits but is found more commonly as a result of industrial pollution. Cr(VI) is much more bioavailable to aquatic organisms than Cr(III), therefore in aquatic ecosystems, Cr(VI) exposure poses a significant threat to aquatic life [47]. The bioavailability of Cr and the rates and routes of its accumulation in aquatic organisms are critical for understanding Cr toxicity and its risk assessment for aquatic organisms and for public health [48]. In this work, we evaluated the effects of exposure to different concentrations in the nM order of chromium (VI) (1, 10, 100 nM) on the properties of protamine-like proteins and the expression levels of their genes and of some stress genes in the gonads of *Mytilus galloprovincialis*. This was done to examine whether even at these doses of chromium (VI) there were any adverse effects on spermatogenesis in this organism, since reproductive capacity is the basis for the survival of the species. A concentration of 1 nM chromium (VI) is normally present in marine waters [33]. Values of 10 and 100 nM were tested to assess the effects of potential increases in chromium concentrations in the sea, given the large anthropogenic pollution by this metal. After exposure of mussels for 24 h to the three different concentrations of chromium (VI), PLs were extracted from mussel spermatozoa and analyzed by both AU-PAGE and SDS-PAGE. PL from mussels unexposed and exposed to chromium did not show significant differences by AU-PAGE, while, by SDS-PAGE a co-migration of PL-II and PL-III was observed, particularly after 1 nM chromium (VI) exposure. The observed co-migration of PL-II with PL-III could be due to a “metal gel shift” phenomenon. This phenomenon was already reported for the hsp protein following the addition of Ni2+, which caused a conformational change of the protein and an alteration of its migration in SDS-PAGE [49]. In addition, the faster migration of the PL-II protein, observed in the SDS-PAGE, was already reported after the exposure of *M. galloprovincialis* to CdCl_2_ [29] and HgCl_2_ [31]. The differences found in the electrophoretic pattern of PLs from mussels unexposed and exposed to chromium, prompted an investigation into their ability to bind and protect DNA from oxidative damage. To this aim, EMSA and protection assays were conducted using both a plasmid DNA and genomic DNA extracted from spermatozoa of unexposed mussels. Additionally, in this case the difference with respect to unexposed mussels was found after the exposure to 1 nM chromium (VI). Indeed, at this exposure dose, more protein was required to reach DNA saturation as shown by EMSA. This assay showed that DNA saturation was not reached even at the wt/wt protein/DNA ratio of 1.8, unlike that achieved with PLs from unexposed mussels and exposed to 10 and 100 nM chromium (VI) for which plasmid DNA saturation was reached at the wt/wt protein/DNA ratio of 0.8. EMSA also showed that the extracts of PL proteins (containing PL-II, PL-III, and PL-IV) from unexposed and exposed mussels interacted with DNA in “all or nothing” mode [50], as sperm H1 histones and *C. variopedatus* PL protein [51,52]. Similarly, with respect to binding of PLs to genomic DNA extracted from unexposed mussels, differences were found in mussels exposed to 1 nM chromium (VI). Given the impaired ability of PLs from mussels exposed to 1 nM chromium VI to bind DNA, we evaluated whether this could affect the capacity of the proteins to protect DNA from oxidative damage in the presence of H_2_O_2_ and CuCl_2_. In fact, although the toxicity mechanisms of heavy metals are not well understood, some common mechanisms underlying their toxicity have been recognized. The production of reactive oxygen species (ROS) and oxidative stress are the major strategies that result in the alteration of proteins, lipid peroxidation, and DNA damage [37]. In addition, sperm DNA fragmentation is considered a major cause of infertility, and the sperm nuclear basic proteins have the main role of complexing the DNA of the spermatozoa in a correct manner in order to prevent oxidative damage [53,54]. Consistent with what we observed in EMSA assays, using both pGEM3 plasmid DNA and genomic DNA, we found a reduced ability to protect DNA with PLs from mussels exposed to 1 nM chromium (VI) compared with unexposed mussels. This suggests that these proteins, having an altered binding to DNA have a reduced ability to protect from free radical damage. Given these results, we then attempted to understand the nature of the interactions between chromium (VI) and PLs. For this aim, we used a hydrophobic dye such as ANS [55] to investigate the structural changes induced to PLs by nM concentration of chromium (VI). ANS is a well-known fluorescent dye specific for binding to the hydrophobic domains of proteins and whose quantum yield greatly depends on the hydrophobicity of the microenvironment around the ligand binding site. In absence of amino acid residues liable of the intrinsic fluorescence in the PLs structures, such as tryptophans, ANS can act as a sensitive reporter of changes in the protein structure, helping in the characterization of the hydrophobic sites present on the protein [56]. As expected, ANS increase fluorescence emission in presence of the PLs mixture, supporting the binding of the probe with the three PLs or their oligomeric complexes. The addition of chromium (VI) concentrations significantly decreased the fluorescence emission of ANS-PLs complex, suggesting a possible reduction of the exposed hydrophobic sites on the PLs, or a competition of the metal with the ANS for the same binding site within the PLs structures, thus being capable to displace the hydrophobic probe from the binding site on the PLs. The degree of ANS displacement was higher at lower chromium (VI) concentrations, while concentrations > 2.5 nM had less impact on ANS-PLs interactions. However, at about 1 nM chromium (VI), we observed an increase of the fluorescence emission of ANS-PLs complexes contextually to a blue shift (Figure 8c,d) in the maximum wavelength of fluorescence emission, afterwards, the fluorescence emission slowly decrease towards a plateau. A possible hypothesis to explain these results have to consider that at very low concentration, less than 1 nM of chromium (VI), specific interactions could be take place between the metal and the structures of PLs, strongly affecting the proteins structures or their oligomeric complexes, not excluding a possible conformational change. Instead, the increased concentration of metal could result in more general electrostatic effects nullifying the specific ones. Being the structures of PLs strongly related to their function, this hypothesis could also explain the evidence obtained with respect their binding to DNA; moreover, the hypothesis of a specific binding of the metal in the PLs structures support a change in the superficial charges that could affect the binding to SDS and the migration in SDS-PAGE. Although these data are still preliminary and more deep studies could be carried on for the analysis of PLs structures, these observations open interesting questions on the specific effect of metals like chromium (VI) on proteins. Thus, as future perspective, we plan to perform the kinetics of release of sperm nuclear basic proteins from sperm nuclei by means of a gradient of NaCl. This experimental approach will allow us to have more information about possible alterations in the binding of these proteins to DNA, because altered release of PL proteins from DNA would indicate abnormal binding to DNA, which in turn would result in incorrect chromatin structure and even difficulties at the time of fertilization. Consistent with the alterations found in the properties of PL proteins, after exposure to 1 nM chromium (VI), we found a very low motility score. At present, we have not yet conducted accurate studies on the morphology of mussel spermatozoa after exposure to these doses of chromium (VI) because at least one TEM analysis is required to properly observe mussel spermatozoa due to their small size. Therefore, this represents a limitation for our results. These analyses require collaboration with experienced morphologists, so this will be another future perspective of our research. With the available data, we can only speculate that the lower DNA binding affinity of PL proteins observed after the 1 nM Cr(VI) exposure dose might produce a defective sperm chromatin structure, which in turn might lead to alterations in sperm morphology. In this work we also evaluated, by RTqPCR the effect of exposure to chromium (VI) on gonadal *mt10* and *hsp70* and PL encoding genes. This because the literature reports an alteration in *mt10* gene expression in *Mytilus galloprovincialis* digestive gland after exposure to metals such as copper, zinc, mercury and cadmium [57]. On the other hand, in our previous work, we observed that exposure to different metals causes alterations in *mt10* and *hsp70* expression in *Mytilus galloprovincialis* spermatozoa and gonads. For example, mercury causes an increase in *mt10* gene expression in *Mytilus galloprovincialis* spermatozoa after exposure of mussels to 10 pM and 100 pM HgCl_2_ [30]. Regarding stress genes, we found that all doses of chromium (VI) exposure led to overexpression of *mt10*, and the highest level of overexpression of this gene was observed just after 1 nM of chromium (VI) exposure. In contrast, for *hsp70*, we found no significant changes in its expression after exposure to chromium (VI). RTqPCR analyses to assess any alterations in the expression of *PL-II*/*PL-IV* and *PL-III* genes in the gonads of mussels exposed to chromium (VI) showed a hypo-expression of *PL-II*/*PL-IV* genes in all three conditions of exposure compared to unexposed mussels. Additionally, in this case, the greatest differences were found for exposure to 1 nM chromium (VI). For the *PL-III* gene, instead, we found no significant changes for the three exposure conditions compared to unexposed mussels. Analyzing all the data obtained, the condition of exposure to chromium (VI) 1 nM appears to be the one that causes the greatest number of alterations at the protein and gene levels. It is also intriguing to note that as chromium (VI) concentration increases, but particularly at the 100 nM value, the ability of PL to both bind and protect DNA from oxidative damage is restored. In addition, at this dose of chromium (VI) exposure, there were no greater changes in the expression of *mt10* than in unexposed mussels. Thus, low concentrations of chromium (VI) appear to induce greater stress in *Mytilus galloprovincialis* gonads and PL proteins properties than the higher concentrations of the same metal tested in this work. This result might suggest a possible hormesis effect. Hormesis is a dose/response relationship characterized by a biphasic effect. Many biological organisms/systems, exposed to a wide range of stimuli, produce opposite responses depending on the dose [58]. We previously found a similar phenomenon in *Mytilus galloprovincialis* following exposure to NiCl_2_ [59]. Incidentally, this phenomenon is also found in plants such as for *Lonicera japonica Thunbriporta* induced by cadmium on growth and photosynthetic performance [60]. In conclusion, the biological relevance of this work is to have shown that even exposure to very low doses of chromium (VI) in the order of nanomolar and in particular 1 nM, un-like 10 and 100 nM, induce adverse effects on spermatogenesis in *Mytilus galloprovincialis* because affects the DNA binding of PLs that are in charge of the proper compaction of the sperm chromatin and consequently the protection of DNA from oxidative damage. Considering the alterations found in the properties of PL following exposure to Cr(VI) in *Mytilus galloprovincialis*, it would be interesting in the future to evaluate any conformational changes in these proteins following exposure of mussels to these doses of chromium (VI) by further fluorescence spectroscopy and circular dichroism experiments. The preliminary data we obtained from fluorescence spectroscopy measurements are encouraging and thus prompt further investigation. As a further perspective, it will be interesting to make measurements using the SEC-MALS technique, which allows the determination of molar mass and size in solution, to highlight possible PL aggregates that could be formed following exposure of mussels to these doses of chromium (VI). Finally, to understand with certainty if these doses of chromium (VI) can induce alterations in the fertilizing capacity of spermatozoa, in vitro fertilization experiments have been planned.

## Figures and Tables

**Table 1:** List of forward and reverse primers used for amplification of the genes analysed and for *GAPDH* gene used as housekeeping gene reference.

Gene	F-Primer	F-Primer Length	R-Primer	R-Primer Length	Accession Number
*GAPDH*	CTGCACCACCAACTGCTT	18	TTCTGGGTGGCAGTGATG	18	SY171038758-018/019
*Hsp70*	CGCGATGCCAAACTAGACAA	20	TCACCTGACAAAATGGCTGC	20	AY861684
*Mt10*	GCCTGCACCTTGTAACTGTAT	21	CTGTACACCCTGCTTCACAC	20	AY566248
*PL-III*	CACCCAACAAGAAGGATGCC	20	CCTTGCCCTTTTCTTTCCCC	20	SY140930274
*PL-II*/*IV*	AAGCCCAAGTAGACGTTCCA	20	TCCGAGGTGTGATGTGTTGA	20	SY140930274

**Table 2:** Mean values of sperm motility, evaluated according to a sperm motility score. Measurements were made on sperm suspensions from *M. galloprovincialis* unexposed and exposed to 1, 10, and 100 nM chromium (VI).

	Score
Unexposed	5
1 nM Cr(VI) exposed	1
10 and 100 nM Cr(VI) exposed	3

## Data Availability

Not applicable.

## Abbreviations

APS	Ammonium persulfate
ANS	8-anilino-1-naphthalenesulfonic acid
ASW	Artificial sea water
AU-PAGE	Acetic acid-urea polyacrylamide gel electrophoresis
SDS-PAGE	Sodium dodecyl sulphate–polyacrylamide gel electrophoresis
EDTA	Ethylenediaminetetraacetic acid
EMSA	Electrophoretic mobility shift assay
PCA	Perchloric acid
PL	Protamine-like
RT-qPCR	Reverse transcript quantitative polymerase chain reaction
*C. variopedatus*	*Chaetopterus variopedatus*
ROS	reactive oxygen species
